# Milk fat miRNome changes in response to LPS challenge in Holstein cows

**DOI:** 10.1186/s13567-023-01231-4

**Published:** 2023-11-22

**Authors:** Christine Leroux, Matteo Cuccato, Karol Pawłowski, Francesca Tiziana Cannizzo, Paola Sacchi, José A. A. Pires, Yannick Faulconnier

**Affiliations:** 1 INRAE, Université Clermont Auvergne, VetAgro Sup, UMR Herbivores, 63122 Saint-Genès-Champanelle, France; 2 Dipartimento di Scienze Veterinarie, Università degli Studi di Torino, Largo Paolo Braccini 2, 10095 Torino, Italy; 3 Department of Pathology and Veterinary Diagnostics, Faculty of Veterinary Medicine, Warsaw Univeristy of Life Sciences, Nowoursynowska 159c, 02-776 Warsaw, Poland

**Keywords:** miRNomes, milk fat, dairy cow, inflammation, LPS

## Abstract

Mastitis is an inflammatory disease in dairy cows, causing economic losses and reducing animal welfare. In order to contribute for the discovery of early and noninvasive indicators, our objective was to determine the effects of a lipopolysaccharide (LPS) challenge on the microRNA profile (miRNome) of milk fat, using microarray analyses in cows. Cows were fed a lactation diet at ad libitum intake (*n* = 6). At 27 ± 3 days in milk, cows were injected with 50 µg of LPS *Escherichia coli* in one healthy rear mammary quarter. Milk samples were collected just before LPS challenge (LPS−) and 6.5 h after LPS challenge (LPS +) from the same cows. Microarray analysis was performed using customized 8 × 60 K ruminant miRNA microarrays to compare LPS− to LPS + miRNome. In silico functional analyses were performed using OmicsNet and Mienturnet software. MiRNome comparison between LPS− and LPS + identified 37 differentially abundant miRNAs (q-value ≤ 0.05). The predicted target genes of the 37 differentially abundant miRNAs are mostly involved in cell life including apoptosis, cell cycle, proliferation and differentiation and in gene expression processes. MiRNome analyses suggest that miRNAs profile is related to the inflammation response of the mammary gland. In conclusion, we demonstrated that milk fat might be an easy and rapid source of miRNAs that are potential indicators of early mastitis in cows.

**Supplementary Information:**

The online version contains supplementary material available at 10.1186/s13567-023-01231-4.

## Introduction

In dairy cows, mastitis is one of the most prevalent inflammatory diseases which affects animal health and welfare, and impacts profitability [1]. Dairy cows are particularly susceptible to mammary gland inflammation during early lactation [2]. In early lactation, cow health is impacted by many factors including metabolic stress, hypocalcemia, and metritis [3, 4] that influence immune function [5]. One aim of the One Health concept is to reduce the use of antibiotics, by prioritizing disease prevention, and promoting early detection. The identification of early indicators for rapid and accurate detection of mastitis could lead to earlier and more effective treatment allowing the animal to recover faster, and therefore reducing the associated economic losses. A better understanding of the molecular regulation of the mammary response to inflammation would allow the identification of such indicators. MicroRNAs (miRNAs) are small (18–25 nucleotides) non-coding RNAs and known to regulate many biological processes including those involved in immune system activity. During disease, differently expressed miRNAs are used as biomarkers of pathological condition [6]. Several studies on the effects of inflammation on miRNA profiles have been conducted to decipher the underlining molecular mechanisms of mastitis. For instance, in the bovine mammary gland tissue, 29 miRNAs were modulated during spontaneous mastitis [7]. Mastitis caused by *Staphylococcus aureus* [8] and *Streptococcus agalactiae* [9] modified the expression of 77 and 35 known miRNAs, respectively. Similarly, the miRNA profile of mammary glands infected by Gram-positive (*S. aureus*) and Gram-negative (*Escherichia coli*) bacteria showed a different response, with 82 and 108 differentially expressed miRNAs, respectively [10]. These studies provided evidence of inflammation effects on miRNA mammary expression. However, the use of mammary tissue biopsies to investigate these molecular regulations requires invasive procedures and is not suitable for large-scale studies. In contrast, liquid sampling is increasingly used as a noninvasive method for diagnosing diseases such as cancer [11, 12] or metabolic disorders [13]. The presence of miRNAs in milk offers a new opportunity to research their use in disease diagnostic [14]. Sun et al. [15] identified 14 known miRNAs differentially abundant in milk extracellular vesicles after *S. aureus* infection, and Lai et al. [16] detected 25 known miRNAs differentially abundant in whey milk of cows with mastitis. Milk fat (MF) globules, secreted by mammary epithelial cells, are abundant in milk and easy to obtain. During their secretion, MF globules load cytoplasmic crescents with miRNAs. Thus, MF is an easily accessible source of miRNAs [17, 18]. In addition, MF miRNomes were reported to portray mammary gland miRNomes more accurately than the other milk fractions [17, 18].

Early lactation cows experience negative energy balance and profound shifts in metabolic and hormonal status. This period is also characterized by increased risk of inflammatory diseases such as mastitis, and altered immune function [2, 19]. In addition, this period of negative energy balance influenced mammary gland gene expression, which may be related to affect the response to inflammation [20]. With the ultimate objective to explore the MF as a source of noninvasive biomarkers of mastitis in dairy cows, the aims of this study were: (1) to determine the effects of a lipopolysaccharide (LPS) challenge, used as a model of Gram-negative bacteria-associated mastitis, on the miRNome of MF; (2) to assess the potential biological functions of the differentially expressed miRNA.

## Materials and methods

### Experimental design: animals and sampling

Six multiparous Holstein cows from the experimental Herbipôle Unit of INRAE Research Center of Auvergne-Rhone-Alpes [21] with a lactation number ranging from 2 to 4 were used. The procedures were approved by the regional ethics committee on animal experimentation (APAFIS #2018062913565518).

Cows were allowed ad libitum intake of a lactation diet containing corn silage (29%, dry matter basis), corn (24.2%), grass silage (25.5%), soybean meal (16.9%) and a vitamin and mineral complement (0.9%). At 27 ± 3 days in milk, one healthy rear mammary quarter of all cows was injected with 50 µg of LPS *E. coli* 0111:B4; (LPS-EB Ultrapure, InvivoGen, San Diego, CA, USA) diluted in 10 mL of sterile saline CDM (Lavoisier, Paris, France) containing 0.5 mg/mL BSA cell culture grade, endotoxin-free, A9576, (Sigma-Aldrich, St. Louis, MO, USA), as described in Pires et al. [19]. Residual milk in mammary gland was collected after AM milking, just before infusion (LPS-) and the end of PM milking, corresponding to 6.5 h after LPS injection (LPS +). This sampling timeline was adopted to target the first step of inflammatory state. The effects of LPS were monitored to confirm the inflammation response of the mammary gland. The inflammation response to LPS was confirmed by multiple indicators, including increased rectal temperature, and milk somatic cell counts, selected milk cytokines, and decrease in milk yield, as previously reported [19].

### RNA preparation

Total RNA from residual milk samples, collected immediately after standard milking, was extracted as previously described [22]. Briefly, immediately after centrifugation at 2000 × *g* for 10 min at 4 °C of fresh residual milk samples, cells and cellular debris were on the bottom of the tubes, MF was collected on the top layer. One g of MF was mixed with 2.0 mL of TRIzol LS solution (Invitrogen Life Technologies Inc.), then 600 µL of chloroform were added before centrifugation at 12 000 × *g* for 20 min at 4 °C. The upper aqueous layer was precipitated using 500 µL of isopropanol overnight. The pellet obtained by centrifugation at 12 000 × *g* for 10 min at 4 °C was washed and RNA was dissolved in 50 μL of RNase-free water. RNAs were stored at − 80 °C until miRNA analyses.

### Microarray analyses

Customized 8 × 60K miRNA microarray (Agilent Technologies, Inc. Santa Clara, USA) were used. This microarray contained 786, 276 and 105 miRNAs from bovine, caprine and ovine sequences, respectively. All the sequences were spotted at least twice. Twelve microarrays were carried out. Total RNA (100 ng) was labelled with Cy3 using the one-color Quick Amp Labeling Kit (Agilent Technologies, Inc. Santa Clara, USA) and hybridized overnight to the microarrays using the High-RPM Gene Expression Hyb Kit (Agilent Technologies, Inc. Santa Clara, USA) following manufacturer’s instructions. After washing, microarrays were scanned using the Innoscan (Innopsys, France) and the resulting TIFF images were processed using Feature Extraction software Version 11 (Agilent Technologies, Inc. Santa Clara, USA). The data are accessible through the GEO series accession number GSE229476. Normalized data obtained with 75th percentile shift date from the microarray assay were analyzed using GeneSpring software (Agilent Technologies, Inc. Santa Clara, USA). We considered the *q*-value described as a powerful approach [23] to control the false discovery rate (FDR), which were obtained using the Storey method [24] with curve fitting model and considered significant at the FDR *q*-value ≤ 0.05.

### In silico functional analyses

In silico functional annotations of miRNAs and their predicted targets were performed using OmicsNet tools [25]. Due to the higher level of annotations in human than in bovine species, we used human tools with miRTarBase (V8.0) experimentally validated miRNA-gene interactions. OmicsNet analyses also identified functional biological categories related to differentially expressed miRNAs, using Panther Biological Processes database by selecting only validated miRNA-gene interactions. Then, we categorized and grouped manually biological processes. The gene lists were also filtered by previously obtained results using Mienturnet software [26]http://userver.bio.uniroma1.it/apps/mienturnet/. The potential miRNAs-genes networks were identified and visualized using Cytoscape (Version 3.8).

## Results

### Effects of LPS challenge on milk fat miRNomes

**Figure 1:**
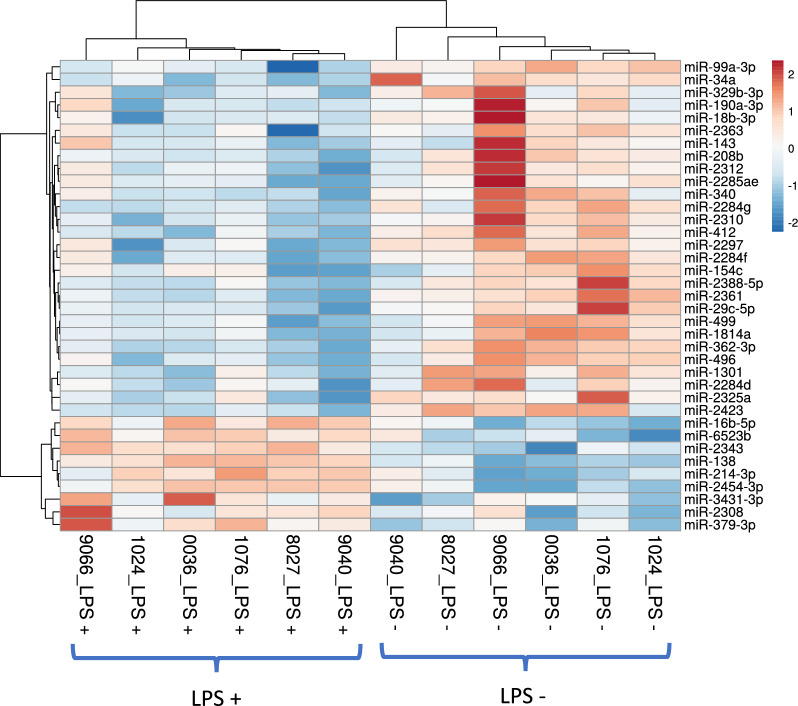
**Heatmap analysis of differentially abundant miRNAs after LPS challenge in early lactation cows by comparing LPS- (just before LPS injection) to LPS+ (6.5 h after 50 µg of LPS *E. coli* injection).** The scale indicated the level of abundance in LPS+ vs. LPS-. Each cow number is indicated at the bottom of the figure.

Before studying the effects of LPS on MF miRNomes, the effect of inflammation was confirmed by increased rectal temperature and, increased milk somatic cell counts. In addition, foremilk IL-8, IL-1β, TNF-α, and CXCL3 concentrations increased in response to LPS (Additional file 1) as previously reported [19, 20]. The comparison between miRNomes of MF obtained before (LPS-) and after (LPS+) revealed 37 differentially abundant miRNAs (DAMs) (Table 1). Among the 37 DAMs, 28 and 9 were downregulated and upregulated, respectively. The heat map analysis of DAMs showed a classification of cows according to the LPS challenge status (Figure 1).

**Table 1:** **Differentially abundant miRNAs (q-value ≤ 0.05) in milk fat after LPS challenge in early lactation cows**

Gene name	q-value	FC
*miR-16b-5p*	0.04	1.15
*miR-18b-3p*	0.05	−1.19
*miR-29c-5p*	0.05	−1.22
*miR-34a*	0.05	−1.21
*miR-99a-3p*	0.04	−1.26
*miR-138*	0.04	1.36
*miR-143*	0.05	−1.18
*miR-154c*	0.05	−1.10
*miR-190a-3p*	0.05	−1.14
*miR-208b*	0.05	−1.38
*miR-214-3p*	0.05	1.24
*miR-329b-3p*	0.04	−1.15
*miR-340*	0.05	−1.21
*miR-362-3p*	0.03	−1.29
*miR-379-3p*	0.04	1.26
*miR-412*	0.04	−1.23
*miR-496*	0.04	−1.36
*miR-499*	0.04	−1.20
*miR-1301*	0.04	−1.27
*miR-1814a*	0.05	−1.33
*miR-2284d*	0.04	−1.19
*miR-2284f*	0.05	−1.24
*miR-2284 g*	0.05	−1.30
*miR-2285ae*	0.05	−1.14
*miR-2297*	0.05	−1.24
*miR-2308*	0.04	1.13
*miR-2310*	0.04	−1.33
*miR-2312*	0.05	−1.18
*miR-2325a*	0.05	−1.23
*miR-2343*	0.05	1.25
*miR-2361*	0.04	−1.30
*miR-2363*	0.04	−1.15
*miR-2388-5p*	0.04	–1.31
*miR-2423*	0.05	−1.27
*miR-2454-3p*	0.05	1.37
*miR-3431-3p*	0.05	1.13
*miR-6523b*	0.05	1.32

FC: fold change in miRNA abundance in LPS+ vs. LPS- cows.

### Comparison of the effects of different miRNA sources on miRNome responses

**Figure 2:**
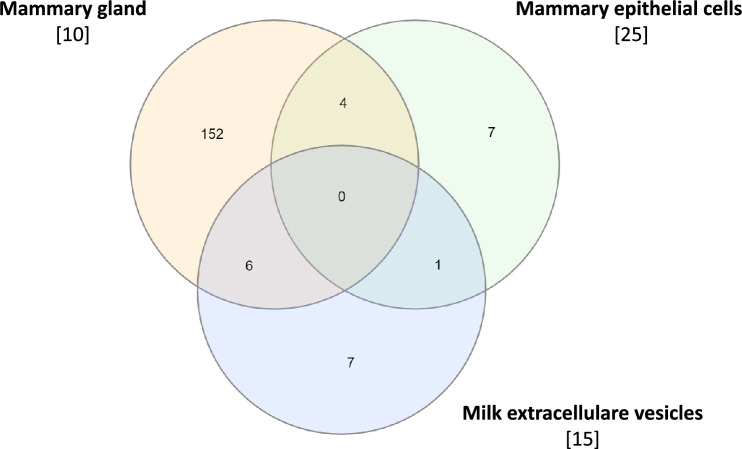
**Venn diagram of the differentially abundant miRNAs after *S. aureus* challenge in mammary gland **[10]**, mammary epithelial cells **[25]**, and milk extracellular vesicles **[15].

**Figure 3:**
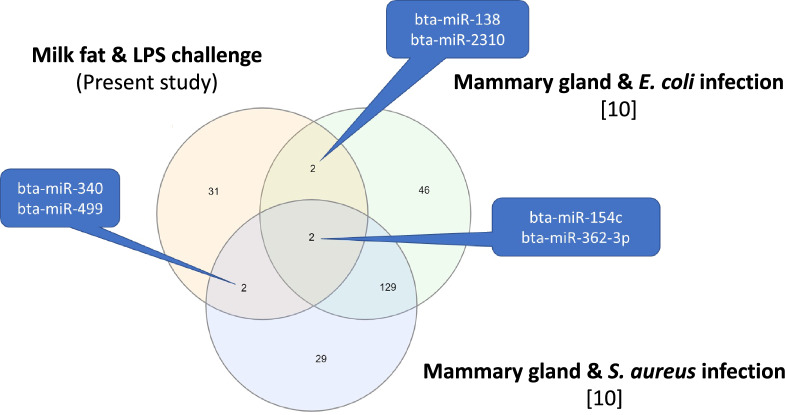
**Venn diagram of differentially abundant miRNAs in milk fat (present study) and mammary gland **[10].

The effects of the miRNAs tissue-sources were evaluated by comparisons of studies using different sources of miRNAs. The effects of *S. aureus* challenge were compared from studies using mammary gland [10], mammary epithelial cells [27] and milk extracellular vesicles [15] as source of miRNAs (Figure 2). We detected no common modulated miRNAs between the three studies of the effects of the same pathogen. Similarly the comparison between the 37 DAMs in response to *E. coli* derived LPS challenge in our study to the 179 differentially expressed miRNAs detected in mammary gland tissue infected with *E. coli* [10] showed only 4 common miRNAs (*miR-154c*, *miR-362-3p*, *miR-138*, and *miR-2310*) with both study (Figure 3). With the aim to identify MF miRNAs as biomarkers of mastitis, we compared the 37 DAMs detected in the present study with those identified by Ju et al. [7] in a study of the effects of spontaneous mastitis on mammary gland miRNome. Two miRNAs (*miR-99a* and *miR-143*) were identified in common between Ju et al. [7] and the present study.

### Functional annotation of the differentially abundant miRNAs

**Figure 4:**
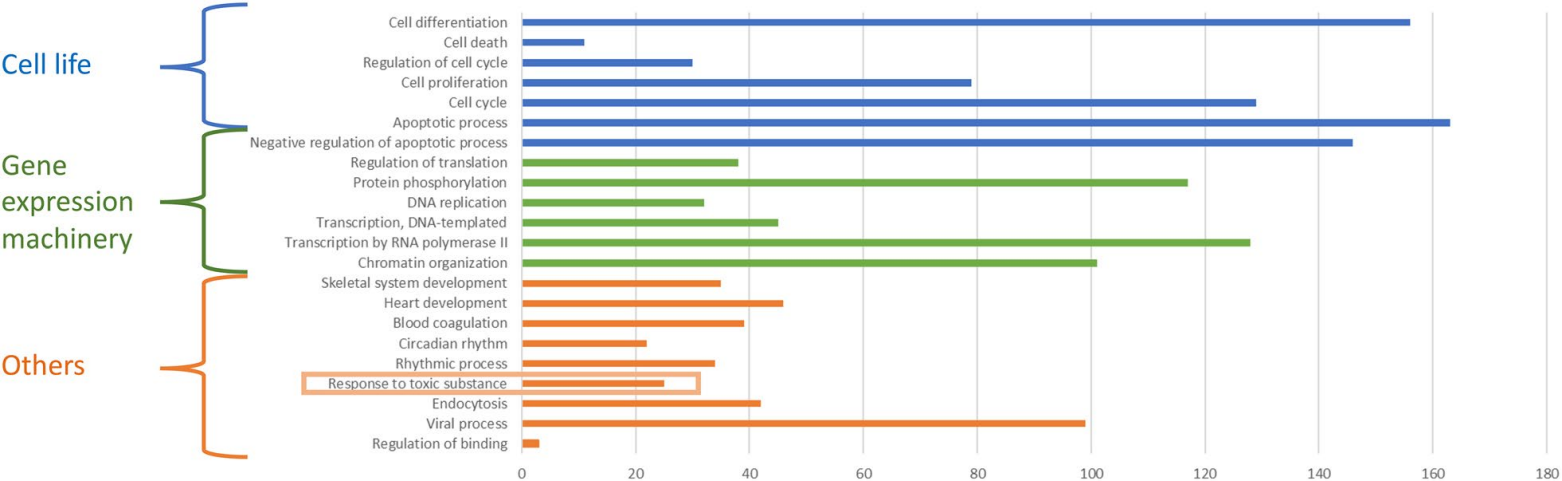
**Functional annotation of the differentially abundant miRNAs in cows identified by OmicsNet **[25]** with Panther database using a threshold *p*-value ≤ 0.1.**

**Figure 5:**
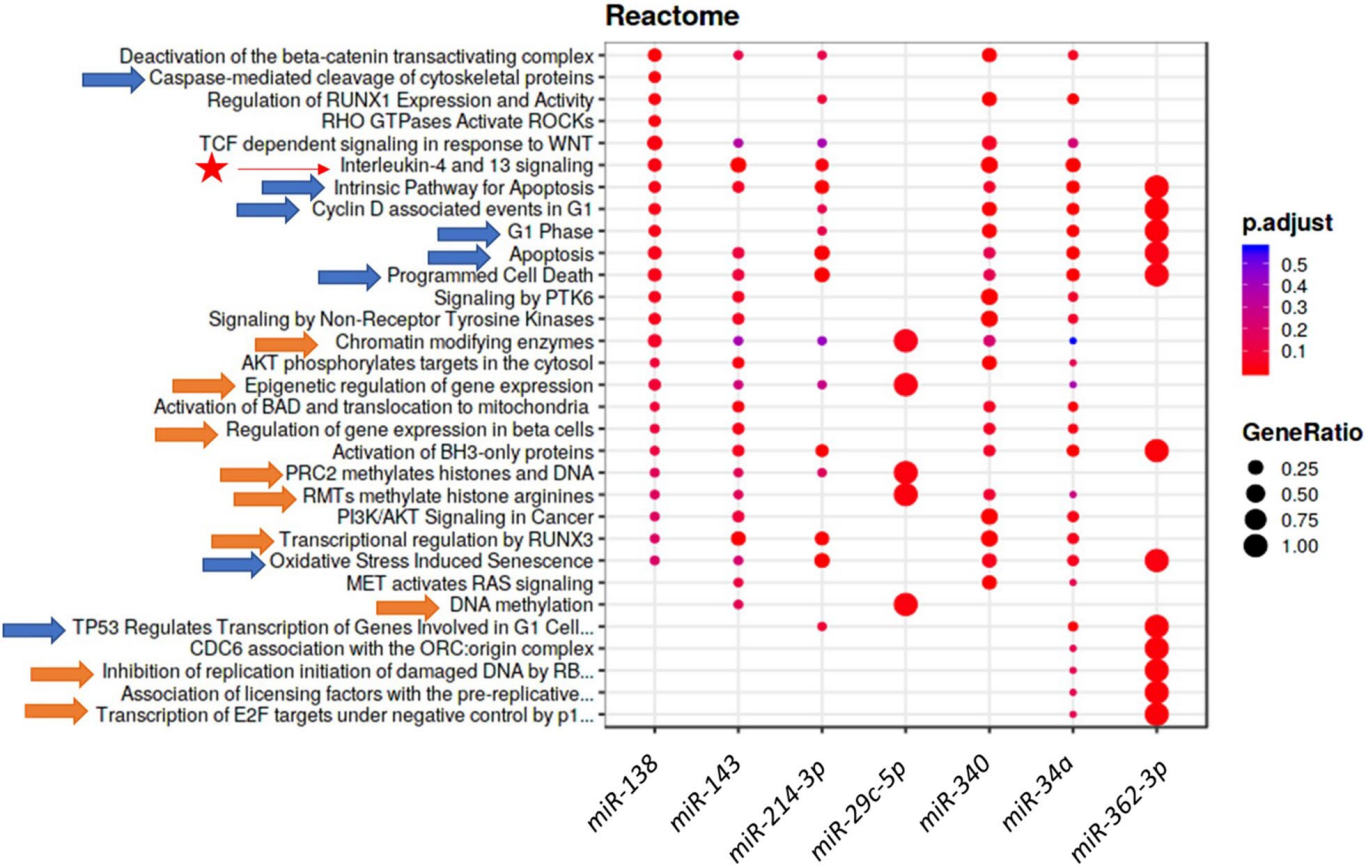
**Reactome analysis of differentially abundant miRNAs in milk fat after LPS challenge identified by Mienturnet software .**[26]. Blue and orange arrows indicate the reactomes related to cell life and to gene expression, respectively, and star pointed out the IL4-13 signaling.

The in silico functional annotation by OmicsNet [25] with Panther database is presented in Figure 4. The targets are mainly involved in cell life (47%) including apoptosis, cell cycle, death, proliferation, and differentiation. The second class of biological processes affected were those involved in gene expression machinery (31%) including chromatin organization, regulation of translation and transcription (Figure 4). One Gene Ontology-Biological Processes (GO-BP) corresponds to a response to toxic substance process (Figure 4). Furthermore, the cell life and gene expression machinery were also highlighted by reactome analysis (Figure 5). Reactome analysis also identified Interleukin-4 and −13 signaling (Figure 5).

**Figure 6:**
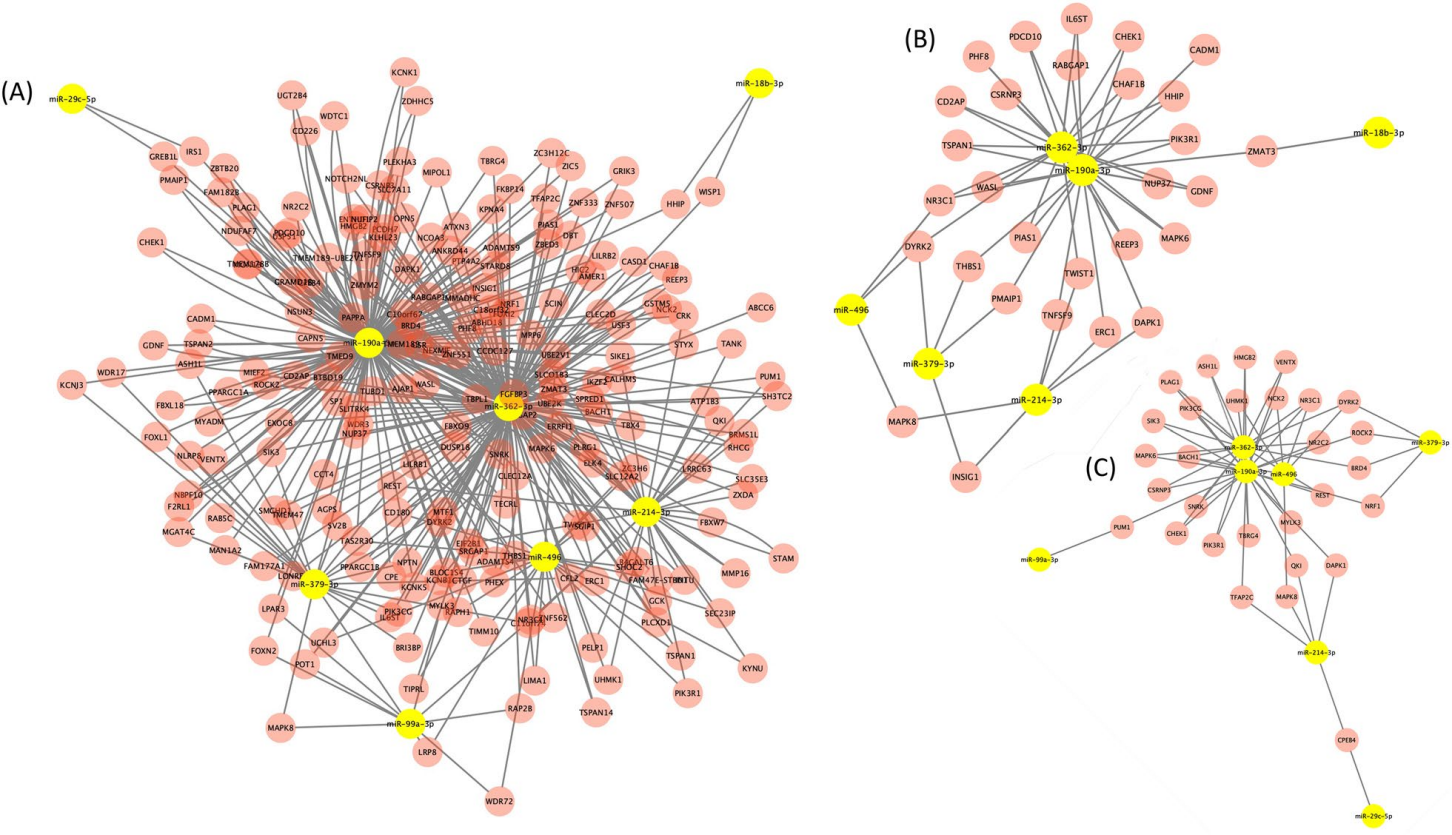
**Networks of the differentially abundant miRNAs in cows identified using Cytoscape**: **A** Total miRNA-gene interactions; **B** miRNA-gene interactions involved in cell life processes; **C** miRNA-gene interactions involved in gene expression machinery.

The network analyses using Cytoscape showed networks that are influenced by 8 known miRNAs (*miR-18b-3p*, *miR-29c-5p*, *miR-99a-3p*, *miR-190a-3p*, *miR-214-3p*, *miR-362-3p*, *miR-379-3p*, and *miR-496*) that were affected by LPS injection (Figure 6A). A total of 448 validated miRNA-gene interactions were found. Two large nodes were observed involving *miR-190a-3p* and *miR-362-3p.* A total of 60 (13.4%) of miRNA-gene interactions were related to cell life (Figure 6B) and a total of 29 (13.8%) of miRNA-gene interactions were linked to gene expression processes (Figure 6C). The two main miRNAs (*miR-190a-3p* and *miR-362-3p*), which were downregulated in MF by LPS injection, were both involved in the regulation of many processes, including cell life and gene expression processes.

## Discussion

To mitigate the negative effects of mastitis, its fast and accurate detection may allow earlier and more efficient treatment, enabling the animal to recover quickly and thus reducing the economic losses. Such detection may be possible by identifying early indicators of inflammation in milk. The immune function is influenced by metabolic disorders, which are often associated with the spontaneous negative energy balance of early lactation [4, 5, 28]. A better understanding of the molecular regulation of the mammary response to inflammation would reduce their negative impacts and allow to identify new early biomarkers. Mammary gland miRNomes are modified during mastitis in the bovine [7, 9, 10, 15]. Mammary gland secretes abundantly MF globules, which are one of the major miRNA-carrying compartments in milk [17, 18]. MF miRNomes showed strong similarity with that of mammary gland tissue [17], however, MF globules are not an exact mirror of mammary gland. Indeed, miRNA composition may differ among mammary gland tissue, mammary epithelial cells and milk fat globules [17].

The number of DAMs is relatively low compared to studies conducted on the mammary gland tissue. Luoreng et al. [10] identified 179 and 162 miRNAs using *E. coli* and *S. aureus* infections, respectively and Li et al. [8] highlighted 77 miRNAs from mammary glands infected by *S. aureus*. In addition to the source of miRNAs (MF vs mammary gland), we cannot exclude that even though most milk somatic cells are eliminated during centrifugation, some may be trapped in MF used for RNA extraction. The trapped cells could contribute to miRNA supply, increasing variability. Moreover, the variability may also be due to animal polymorphism, or to the methodology used for miRNome analyses. However, the number of 37 DAMs in the present study is in line with those obtained using different fractions of milk. For instance, in response to *S. aureus* infection, Sun et al. [15] and Lai et al. [16] reported 14 and 25 DAMs in milk extracellular vesicle and in milk whey fraction from cows with clinical mastitis, respectively.

The variability of miRNome responses could be due to the sources of miRNAs and also by a variety of pathogens. In order to evaluate the factors that may affect miRNA abundance in response to inflammation, we compared available published data using different sources of miRNAs to study the effect of *S. aureus* (Figures 2, 3). The miRNome responses after *S. aureus* challenge in mammary gland [10], mammary epithelial cells [27] and milk extracellular vesicles [15] were differents with no common miRNA (Figure 2). The variability of response in these tissues did not allow to detect many common miRNAs among these studies (Figure 2). The comparison between the 37 DAMs in response to *E. coli* derived LPS challenge in our study to the differentially expressed miRNAs detected in mammary gland tissue infected with *E. coli* [10] showed 4 common miRNAs (*miR-154c*, *miR-362-3p*, *miR-138*, and *miR-2310*) with both studies (Figure 3). Among them, *miR-154c*, downregulated in the present study, is known to be stable and highly expressed in the mammary gland [10] and to influence the cell proliferation in melanoma and suppressed cell invasion and migration in nasopharyngeal carcinoma [29]. These roles may be related to the inflammation processes of the mammary gland after the LPS challenge in cows and must be studied more deeply. In addition*, miR-362* has been also described to repress cell proliferation, migration, invasion and epithelial-mesenchymal transition in sinonasal carcinoma [30]. The downregulation of *miR-154c* and *miR-362* in response to LPS injection could help cell migration through mammary tissue and the epithelium remodeling in response to inflammation. The LPS challenge induces recruitment of macrophages, which is potentially facilitated by the downregulation of *miR-362* and *miR-154c*. The influence of differentially expressed miRNAs on the invasion of epithelial cells was also suggested to occur in mammary gland parenchyma during inflammation [31]. To evaluate the effects of different pathogens, we compared studies of *E. coli* and *S. aureus* effects. Luoreng et al. [10] studied the effects of *E. coli* (179 differentially expressed miRNAs) and *S. aureus* (162 differentially expressed miRNAs) on mammary gland miRNomes and showed 131 common miRNAs regulated by infection (Figure 3). Similarly, Jin et al. [27] identified 8 common miRNAs among the 17 and 14 differentially expressed miRNAs in mammary epithelial cells after *E. coli* and *S. aureus*, respectively.

The comparison with a study of spontaneous mastitis [7] showed two miRNAs (*miR-99a* and *miR-143*) in common. These two miRNAs were reported to influence proliferation and apoptosis in cancer models [32, 33]. The decrease of their expression after the LPS challenge in synergy with *miR-154c* and *miR-362-3p* could facilitate apoptosis of inflamed cells. Unfortunately, none of the miRNA was common between the present study and those performed using milk to obtain EVs in a *S. aureus* challenge study [15] and whey fraction during mastitis [16]. This may be due to the inherent variability of both the different source of miRNAs and the different pathogens. All these comparisons showed different responses due to the studied miRNA sources, and, also in a lesser extent, the involved pathogens. These differences should be considered when a set of miRNAs will be used as biomarker of mastitis diagnosis.

In parallel to the identification of potential indicators of mastitis, the in silico functional annotation identified the biological processes potentially regulated by the 37 differently abundant miRNAs in bovine MF in response to the LPS challenge (Figure 4). As expected, the main affected biological process corresponded to cell life (47%) including apoptosis, cell cycle, death, proliferation, and differentiation. The second class of biological processes affected were those involved in gene expression machinery (31%) including chromatin organization, regulation of translation and transcription (Figure 4). One Gene Ontology-Biological Processes (GO-BP) corresponds to a response to toxic substance process. Furthermore, the cell life and gene expression machinery were also highlighted by reactome analysis (Figure 5). In line, network analysis using Cytoscape identified also a total of 60 (13.4%) of miRNA-gene interactions were related to cell life (Figure 6B) and a total of 29 (13.8%) of miRNA-gene interactions were linked to gene expression processes (Figure 6C).

Reactome analysis identified interleukin signaling, which is consistent with an inflammation status and increased cytokine concentrations in milk after LPS injection (Figure 5; Additional file 1). The role of interleukins in mammary epithelial cell response to infection has been previously described [34]. Interactions between miRNAs and several proinflammatory cytokines playing crucial roles in the modulation of inflammatory signaling pathways were previously reported [35].

The network analyses using Cytoscape identified 8 known miRNAs (*miR-18b-3p*, *miR-29c-5p*, *miR-99a-3p*, *miR-190a-3p*, *miR-214-3p*, *miR-362-3p*, *miR-379-3p*, and *miR-496*) that were affected by LPS injection (Figure 6A). A total of 448 validated miRNA-gene interactions were found. Two large nodes were observed involving *miR-190a-3p* and *miR-362-3p.* The two main miRNAs (*miR-190a-3p* and *miR-362-3p*), which were downregulated in MF by LPS injection, were both involved in the regulation of many processes, including cell life and gene expression processes. Indeed, previous studies showed the involvement of *miR-362-3p* in the induction of apoptosis. Indeed, a downregulation of *miR-362-3p* was also detected in mouse microglial cells (BV2) treated with LPS injection and associated with an increased occurrence of apoptotic cells [36]. In addition, *miR-362-3p* was also downregulated in LPS-treated PC12 cells mimicking neurological disorders [37]. The authors suggested a possible role of LPS in the induction of apoptosis and repression of cell proliferation. Other studies reported a role of *miR-362-3p* as a tumor suppressor and an anti-inflammatory activity in several tumors, i.e. cervical adenocarcinoma [38], ovarian [39], renal [40] and human breast [41] cancer. Taken together these results suggested that the downregulation of *miR-362-3p* is involved in the induction of apoptosis. Considering that LPS is a powerful inflammation inducer, our results are in line with the abovementioned studies and consolidate the role of LPS and the downregulation of *miR-362-3p*. The second major miRNA identified by network analysis, *miR-190a-3p* is downregulated by LPS. This miRNA was reported to be an inducer of cell proliferation and migration in glioma [37] and associated with apoptosis induction in the chicken liver [42], which is not in line with the downregulation of this miRNA observed in the present study. However, several studies demonstrated a complex regulation of *miR-190a-3p* and its target genes, mediated by long non-coding RNAs [43, 44]. Therefore, further studies should be conducted to better understand the role of *miR-190a-3p* in the modulation of LPS mammary response. The network of miRNA-target enrichment analysis identified two major MF miRNAs, *miR-190a-3p* and *miR-362-3p*, down regulated by LPS challenge and both involved in the induction of apoptosis. However, their potential role in inflammation must be deeply investigated.

In conclusion, we showed that MF is a suitable source of miRNAs during inflammation challenge and MF miRNome is modified by inflammation challenge in early lactation cows. The 37 DAMs in MF identified from the comparison between LPS- and LPS + Holstein cows, could be used as a set of candidate miRNAs for future studies. These 37 miRNA modifications are detectable rapidly after LPS challenge (6.5 h) and thus could be very useful for early diagnosis of mastitis. However, we also highlighted differences depending on the source of miRNAs and to a lesser extend in the considered pathogens. Thus, the identification of a unique miRNA as a biomarker of inflammation status may not be possible, and a set of miRNAs may be required instead. The set of the 37 miRNAs identified in the present study must be validated by studying different models. Spontaneous mastitis caused by different etiological agents are needed to explore this panel of MF miRNAs as potential biomarkers for the early diagnosis of mastitis.

### Supplementary Information

**Additional file 1: Rectal temperature, milk somatic cell count (SCC), milk tumor necrosis factor (TNF)-α, milk IL-8, milk IL-1β, and milk chemokine (C-X-C motif) ligand 3 (CXCL3) concentrations in response to LPS challenge in early-lactation cows1. **

## Data Availability

The dataset supporting the conclusions of this article is available in the Gene Expression Omnibus (GEO; GSE229476) repository.
