# Supplementary material for: Milk fat miRNome changes in response to LPS challenge in Holstein cows
Source: Vet Res. 2023 Nov 22;54:111. doi: 10.1186/s13567-023-01231-4 (PMC10666322; doi:10.1186/s13567-023-01231-4)
Supplement: Supplementary file 1 — Additional file 1: Rectal temperature, milk somatic cell count (SCC), milk tumor necrosis factor (TNF)-α, milk IL-8, milk IL-1β, and milk chemokine (C-X-C motif) ligand 3 (CXCL3) concentrations in response to LPS challenge in early-lactation cows1. [file 13567_2023_1231_MOESM1_ESM.pdf]

Supplemental Table S1. Rectal temperature, milk somatic cell count (SCC), milk tumor necrosis factor (TNF)-α, milk IL-8, milk IL-1β, and milk chemokine (C-X-C motif) ligand 3 (CXCL3) concentrations in response to LPS challenge in early-lactation cows [1].

Item[2]	Time		*P*-value
	Before LPS	After LPS	
Rectal temperature (°C)	38.2 (0.1)	40.8 (0.3)	< 0.001
Milk			
SCC [3]	78 (425)	2019 (425)	< 0.01
TNF-α (pg/mL) [4]	206 (1357)	3897 (1862)	0.06
IL-8 (ng/mL) [4]	0	51.0 (5.5)	< 0.001
IL1-β (pg/mL) [4]	1 (0.7)	498 (97)	< 0.001
CXCL3 (ng/mL) [4]	219 (83)	1724 (133)	< 0.001

[1] Six multiparous Holstein cows were allowed ad libitum intake of a lactation diet throughout the study. At 27 ± 3 (mean ± SD) days of lactation, one healthy rear mammary quarter was injected with 50 µg of LPS (E. coli 0111:B4).

[2] Values are LSM (SEM).

[3] SCC: somatic cell count in whole udder composite milk collected during PM/AM milkings that preceded the LPS injection, and during the PM/AM milking that folowed the LPS injection.

[4] Concentrations in foremilk samples that were collected from the injected mammary quarter before AM milking and LPS challenge, and before the following PM milking, approximately 6 h after LPS injection.
